# Salivary microbiome profiles of oral cancer patients analyzed before and after treatment

**DOI:** 10.1186/s40168-023-01613-y

**Published:** 2023-08-05

**Authors:** Anna I. Mäkinen, Vincent Y. Pappalardo, Mark J. Buijs, Bernd W. Brandt, Antti A. Mäkitie, Jukka H. Meurman, Egija Zaura

**Affiliations:** 1 Department of Oral and Maxillofacial Diseases, University of Helsinki and Helsinki University Hospital, P.O. Box 63, 00014 Helsinki, Finland; 2 Department of Preventive Dentistry, Academic Centre for Dentistry Amsterdam (ACTA), Vrije Universiteit Amsterdam and University of Amsterdam, Amsterdam, the Netherlands; 3 Department of Otorhinolaryngology - Head and Neck Surgery, University of Helsinki and Helsinki University Hospital, Helsinki, Finland; 4 Research Program in Systems Oncology, Faculty of Medicine, University of Helsinki, Helsinki, Finland; 5 Division of Ear, Nose and Throat Diseases, Department of Clinical Sciences, Intervention and Technology, Karolinska Institutet and Karolinska University Hospital, Stockholm, Sweden

**Keywords:** Oral cancer, Cancer treatment, Oral microbiome

## Abstract

**Background:**

Treating oral squamous cell carcinoma (OSCC) introduces new ecological environments in the oral cavity. This is expected to cause changes in the oral microbiome. The purpose of this study was to gain new information on the salivary microbiome of OSCC patients in order to improve the aftercare of OSCC patients. The aims of this study were to investigate possible changes in the salivary microbiome profiles of OSCC patients before and after cancer treatment and to compare these changes with the profiles of healthy controls.

**Patients and methods:**

Paraffin-stimulated whole saliva samples were collected, and the salivary flow rate was measured from 99 OSCC patients prior to surgical resection of the tumor and other adjuvant therapy. After treatment, 28 OSCC patients were re-examined with a mean follow-up time of 48 months. In addition, 101 healthy controls were examined and sampled. After DNA extraction and purification, the V4 hypervariable region of the 16S rRNA gene was amplified and sequenced using Illumina MiSeq. The merged read pairs were denoised using UNOISE3, mapped to zero-radius operational taxonomic units (zOTUs), and the representative zOTU sequences were assigned a taxonomy using HOMD. Descriptive statistics were used to study the differences in the microbial profiles of OSCC patients before and after treatment and in comparison to healthy controls.

**Results:**

At baseline, the OSCC patients showed a higher relative abundance of zOTUs classified as *Streptococcus anginosus*, *Abiotrophia defectiva*, and *Fusobacterium nucleatum*. The microbial profiles differed significantly between OSCC patients and healthy controls (*F* = 5.9, *p* < 0.001). Alpha diversity of the salivary microbiome of OSCC patients was decreased at the follow-up, and the microbial profiles differed significantly from the pre-treatment (*p* < 0.001) and from that of healthy controls (*p* < 0.001).

**Conclusions:**

OSCC patients’ salivary microbiome profile had a higher abundance of potentially pathogenic bacteria compared to healthy controls. Treatment of the OSCC caused a significant decrease in alpha diversity and increase in variability of the salivary microbiome, which was still evident after several years of follow-up. OSCC patients may benefit from preventive measures, such as the use of pre- or probiotics, salivary substitutes, or dietary counseling.

Video Abstract

**Supplementary Information:**

The online version contains supplementary material available at 10.1186/s40168-023-01613-y.

## Introduction

In 2020, there were an estimated 377,700 new cases of lip and oral cavity cancers worldwide with over 177,700 new deaths, making it the 16th most common cancer in the world [1, 2]. The suboptimal results regarding both survival and treatment-related side effects warrant further research.

Management of oral squamous cell carcinoma (OSCC) is based on surgical resection of the tumor with or without adjuvant treatment, i.e., radiotherapy or chemoradiotherapy [3]. The surgical approach often includes reconstruction of the oncological defect [4]. These treatment approaches frequently lead to functional deficits in chewing or speaking, as well as dry mouth (xerostomia). Lymph nodes at risk for metastases are treated either with neck dissection or by radiotherapy [5], which may both affect the salivary glands and further decrease salivary flow. These changes lead to a new ecological environment in the oral cavity, which is expected to also cause changes in the oral microbiome.

Associations between microbial dysbiosis and different gastrointestinal cancers have been previously studied [6–8]. The role of microbial dysbiosis in OSCC specifically is also increasingly recognized [9, 10]. Bacteria such as *Capnocytophaga gingivalis, Prevotella melaninogenica*, and *Streptococcus mitis* have been suggested as possible biomarkers for OSCC [11]. Furthermore, several studies have suggested that *Porphyromonas gingivalis* and *Fusobacterium nucleatum* might be promoters of tumorigenesis in OSCC [12–15].

Against this background, the aims of our present study were (1) to compare the salivary microbial profiles of OSCC patients with those of healthy controls and (2) to study the possible changes in the salivary microbial profiles from before cancer treatment to the post-treatment phase. We hypothesized that the salivary microbial profiles of the patients differ from those of the controls. Further, we anticipated that cancer treatment affects the microbiome causing the microbial profiles to differ between the pre-treatment and post-treatment samples.

## Patients and methods

This study is part of a larger project on oral cancer that examines 100 oral squamous cell cancer patients at the beginning of their treatment path and 44 patients at follow-up [16]. Additionally, 103 age- and sex-matched controls were studied.

### Cancer patients

One hundred patients diagnosed with OSCC and referred for protocol cancer surgery to the Department of Oral and Maxillofacial Diseases, Helsinki University Hospital, Helsinki, Finland, between the years 2011 and 2014, were enrolled in the study and asked to give a saliva sample prior to their cancer surgery. Inclusion criteria were oral cavity cancer of squamous cell origin with planned surgical removal of the tumor. Exclusion criteria were cancer of the lip, tonsils, larynx, and/or pharynx; tumor of origin other than squamous cells; and the patient’s inability to give informed consent. After a follow-up time of at least 19 months, 44 of the patients continued their participation by providing another, post-treatment sample. Sixty-six patients were lost at follow-up due to patient-related factors such as death, unwillingness to continue in the study, or moving outside of the Helsinki University Hospital district area. The medical and dental status of the patients was recorded per routine protocol both at the pre-treatment and post-treatment examinations. Hospital records including basic patient characteristics as well as tumor- and treatment-related data were available for the analyses.

### Controls

One hundred and three age- and sex-matched controls were recruited from the Helsinki University Hospital Department of Otorhinolaryngology—Head and Neck Surgery and the Helsinki Day Activities Centre for the Elderly. The inclusion criteria were age between 35 and 100 years with no previously treated or currently diagnosed cancer of the head and neck area. Data on basic characteristics were recorded using a structured questionnaire including smoking and alcohol use habits, general health status, and denture wearing.

### Sample collection and Candida counts

Paraffin-wax-stimulated whole saliva samples were collected from all participants in a forward-leaning sitting position with a 30-s pre-stimulation by the paraffin-wax chewing followed by 5 min continued stimulation and collection by spitting into a sterile Nunc™ conical test tube (Thermo Scientific™, Thermo Fisher Scientific Inc., Waltham, MA USA). Stimulated salivary flow rate (SFR) was recorded as ml/min, and hyposalivation was determined as SFR below 0.5 ml/min [17]. All samples were stored on ice immediately after collection and delivered to the Department of Oral and Maxillofacial Diseases laboratory at the University of Helsinki, Helsinki, Finland, for cultivation and identification of *Candida* as described in our previous study [16]. Candidiasis was determined as having a salivary *Candida* concentration of over 400 colony-forming units (CFU) per ml [18]. Up to 2 ml of the remaining samples were stored at − 80 °C before delivery in dry ice for microbiome analysis at the Academic Centre for Dentistry Amsterdam (ACTA), Amsterdam, the Netherlands. In total, 236 samples had enough saliva volume left for microbiome studies. Of these, 99 were pre-treatment patient samples, 34 were post-treatment patient samples, and 103 originated from the control subjects.

### Sample processing

#### DNA isolation

DNA isolation was done in batches of 84 samples. To control for potential contaminations, isolation blanks (for kit chemicals) were added to each batch.

The saliva samples were thawed and vortexed extensively, and 200 μl was transferred to an assigned well in a 1.1-ml deep-well plate containing 250-μl 0.1-mm Zirconia beads (BioSpec Products, Inc. Bartlesville, OK, USA), 200 μl of phenol (Rotiphenol, Carl Roth GmbH & Co. KG, Germany), and 100 μl of lysis buffer (MagMini DNA isolation kit, LGC Genomics Ltd., Hoddesdon, UK). After sealing, the deep-well plate was placed in a Mini-BeadBeater-96 (BioSpec Products, Bartlesville, OK, USA) and subjected to four times 2 min bead beating at 2100 oscillations/min.

DNA extraction and purification were done using the MagMini DNA Isolation Kit (LGC Genomics Ltd., Hoddlestone, UK). For measuring the bacterial DNA yield, DNA was subjected to qPCR with universal primers specific to the bacterial 16S rRNA gene [19, 20]. For fungal yield, primers and probe specific to the fungal 28S rRNA gene were used [21].

#### PCR amplification and sequencing

The V4 hypervariable region of the 16S rRNA gene in each sample was amplified using 1 ng DNA with 1 μM of each barcoded forward and reverse primer and performing 30 amplification cycles [22]. As PCR controls, negative PCRs (PCR mix with DNA-free water) were included. After quantification of the DNA concentration of PCR products, samples, isolation controls, and negative PCRs were mixed into an equimolar pool and loaded on an agarose gel. The band containing the amplicon was cut out and cleaned with GE Illustra™ GFX PCR DNA and Gel Band purification kit (Sigma Aldrich, St Louis, USA).

Paired-end sequencing was conducted on the MiSeq platform (Illumina, San Diego, CA, USA) using a MiSeq Reagent kit V3 and 2 × 251 nt at the Core Facility Genomics, Amsterdam UMC (Amsterdam, the Netherlands). The flow cell was loaded with 10 pmol DNA containing 30% PhiX.

Sequence data were processed as described in Kahharova et al. [23]. Briefly, paired-end reads were merged, quality-filtered, and denoised using UNOISE3 [24, 25], after which the sequences were mapped to zero-radius operational taxonomic units (zOTUs). The representative (most abundant) sequence of each zOTU was assigned taxonomy according to HOMD (v.14.51) [26]. The zOTU table was subsampled at 7900 reads/sample, causing the loss of 3 follow-up samples and 2 control samples that did not reach this threshold. Additionally, 3 patients were found to have recurrent cancer at follow-up and were excluded from the analyses; thus, 99 pre-treatment samples, 28 follow-up samples, and 101 control samples were available for statistical analyses.

### Statistical analyses

PAST software version 4.03 [27] was used to calculate the alpha diversity of the samples using untransformed data (see Additional file 1) while the principal component analysis (PCA) and permutational analysis of variance (PERMANOVA, 9999 permutations, Bray–Curtis similarity) tests of the cross-sectional data, i.e., the OSCC patients versus the healthy controls, were calculated using log2-transformed data. On the longitudinal data, i.e., the OSCC patients before and after cancer treatment, a PERMANOVA with restricted permutations on the individual patient and Bray–Curtis distance were calculated using adonis2 (vegan v.2.5–6 [28]; R v.3.6.3 [29]; R Core Team 2020).

For the basic data of both cross-sectional and longitudinal studies, the continuous variables age, SFR, Shannon diversity index, salivary *Candida* concentration (log10[CFU/ml]), and fungal and bacterial loads (ng/μl, 28S, and 16S qPCR, respectively) were tested for normality and differences in means or distribution (independent samples *t* test and independent samples Mann–Whitney *U* for cross-sectional data, related-samples *t* test or paired-samples Wilcoxon signed-rank test for longitudinal data) using IBM SPSS Statistics v.27. For categorical variables sex, smoking, alcohol use, and edentulism, a chi-square test was performed also using SPSS. A PERMANOVA was used to test differences in the microbial profiles of the patients versus the healthy controls as well as comparing the effects of SFR (normal vs hyposalivation), smoking, alcohol use, and edentulism on the microbial profiles of each group separately.

ZOTUs whose abundance differed significantly between the groups were evaluated by linear discrimination analysis (LDA) of effect size (LEfSe) [30], and the results were additionally tested for significance with the Mann–Whitney *U* test (cross-sectional data) and Exact sign test (longitudinal data) using SPSS. Benjamini–Hochberg false discovery rate (FDR) correction of *p* values for the significant zOTUs was done for longitudinal data using R and the FDR was set to 0.05.

Differences in the microbiomes of OSCC patients at baseline and the healthy controls were also analyzed using the Global Test function in R (gt function, globaltest package, v. 5.44.0, [31], see Additional file 1). First, the zOTU data was filtered to include zOTUs present in at least 25% of samples and normalized using the trimmed mean of *M* value (TMM, edgeR package, v.3.32.1 [32]). The relationships between cancer status and the covariates sex, age, edentate status (yes/no), smoking (yes/no), alcohol use (yes/no), SFR, *Candida* concentration (log10[CFU/ml]), and bacterial load (16S rRNA gene qPCR, ng/μl) were tested with chi-square test and the covariate “smoking” was removed from correction as it was a confounding factor to the point of canceling the effect of cancer. Additionally, cases with values “unknown” or “casual” (where applicable) for smoking, alcohol use, and dentition were removed from further analyses. After using the glmFit function (edgeR package, v.3.32.1 [33]) to evaluate the impact of the covariates sex, age, alcohol use, SFR, dentition status, *Candida* concentration, and bacterial load on the microbiome, the Global Test was performed using hyperbolic arcsin (asinh [34]) transformed residuals to assess the relationship between the microbiome and cancer status.

## Results

The basic characteristics of the study population are given in Table 1. The groups were biased with regard to smoking, as the cancer group had more smokers than the control group (chi-square,* p* < 0.001). In the microbiome analyses, the subsampled dataset included 857 zOTUs representing 11 phyla and 137 genera or higher taxa.

**Table 1:** Basic characteristics of OSCC patients at baseline and healthy controls

	OSCC, *N* = 99	Control, *N* = 101	Difference between groups
Sex^a^, male (%)	54 (54%)	46 (46%)	NS
Mean age, years (SD)	68.0 (10.3)	66.4 (14.3)	NS
Smokers (%)	43 (43%)	8 (7.9%)	*p* = 1.6E-08
Alcohol-users (%)	62 (62%)	59 (59%)	NS
Edentate (%)	12 (12%)	5 (5.0%)	NS
Mean SFR ml/min (SD)	1.3 (0.9)	1.4 (0.8)	*p* = 0.036
Dry mouth, SFR < 0.5 ml/min (%)	21 (21%)	14 (14%)	NS
Mean concentration of bacteria, 16S qPCR, ng/μl (SD)	3.5 (3.3)	3.0 (2.3)	NS
Candidiasis^b^ (%)	42 (42%)	40 (40%)	NS
Mean *Candida* concentration, log10 (CFU/ml) (SD)	2.3 (1.2)	2.2 (1.2)	NS
Mean concentration of fungi, 28S qPCR, ng/μl (SD)	0.007 (0.02)	0.003 (0.005)	NS
Tumor class^c^			
T1 (%)	46 (46%)		
T2–T4 (%)	53 (53%)		
Stage^c^			
Stages I–II (%)	57 (57%)		
Stages III–IV (%)	42 (42%)		
Treatment			
Radiotherapy-treated (%)	44 (44%)		
Chemotherapy-treated (%)	5 (5.1%)		

*SFR* salivary flow rate, *SD* standard deviation, *NS* non-significant

^a^Binary categorization determined using social security number

^b^*Candida* growth ≥ 400 colony-forming units (CFU) per milliliter of the saliva

^c^According to the 7th edition of the American Joint Committee on Cancer Staging Manual

### Comparison of patients and controls

**Fig. 1:**
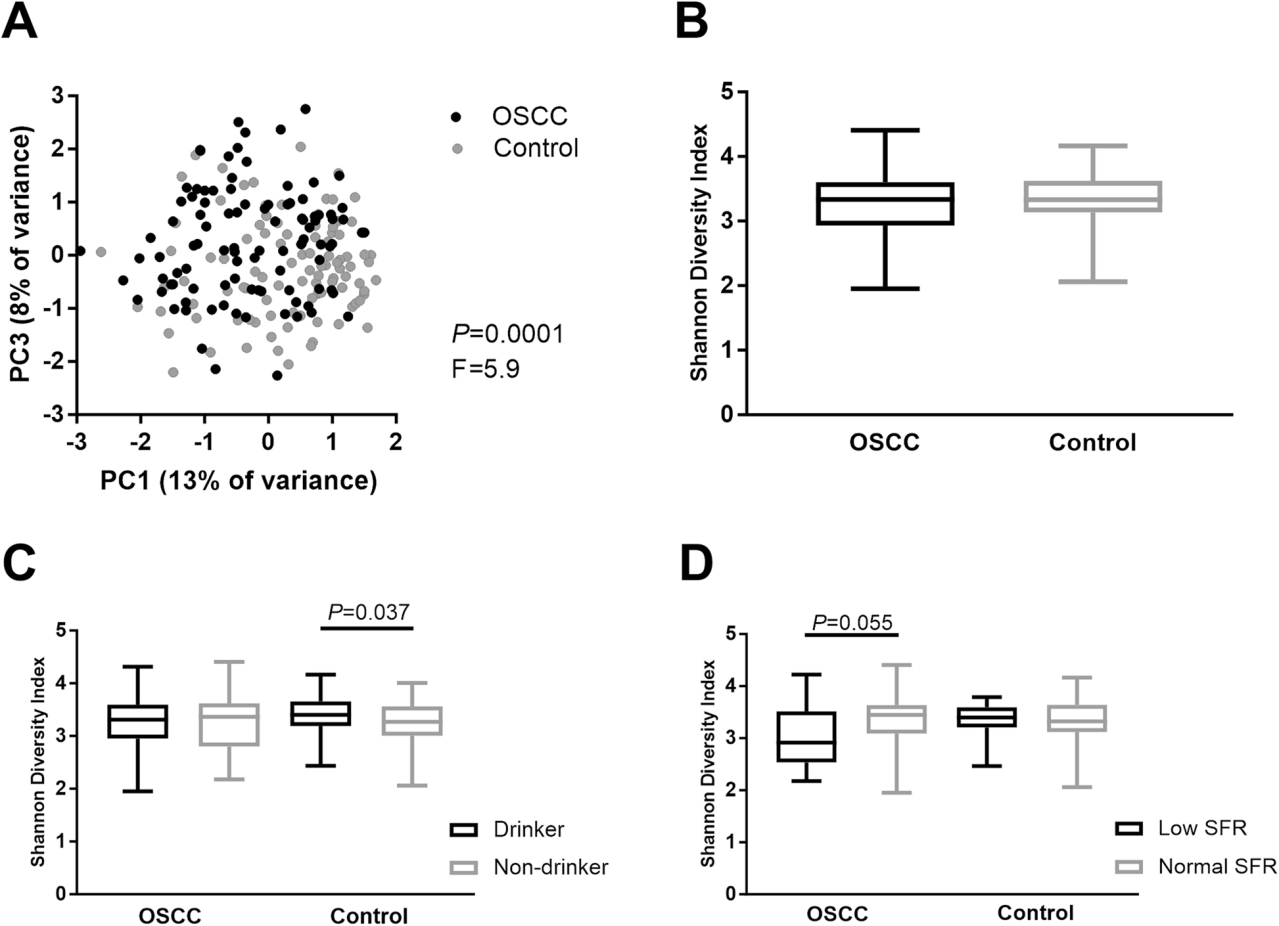
Comparison of OSCC patients and healthy controls. **A** Principal component analysis of all OSCC patients versus healthy controls, *p* = 0.0001, *F* = 5.9—PERMANOVA. **B** Shannon Diversity Index of the OSCC samples versus healthy controls, *p* = non-significant, independent samples *t* test. **C** Shannon Diversity Index in OSCC patients versus healthy controls by alcohol consumption, independent samples *t* test. **D** Shannon Diversity Index of OSCC patients versus healthy controls by salivary flow rate (SFR): low—< 0.5 ml/min, normal—≥ 0.5 ml/min, independent samples *t* test

The subsampled dataset comprised samples from 99 pre-treatment OSCC patients and 101 controls. Figure 1 shows comparisons between the OSCC and control samples. First, all patient’s and control’s zOTU results were studied and compared to each other with no corrections for covariates (“biased” data). The microbial profiles of the two groups differed from each other statistically significantly (PERMANOVA, *F* = 5.9, *p* < 0.001), but no significant difference was found in the Shannon diversity index (independent samples *t* test).

An alcohol-drinking habit had a significant effect on the Shannon diversity index in the control group (independent samples *t* test *p* = 0.037), but not in the OSCC group. Drinkers had a higher bacterial diversity of saliva. The effect of hyposalivation on Shannon diversity was nearly significant for the OSCC group (independent samples *t* test *p* = 0.055), while the difference was not significant in the controls. OSCC patients with hyposalivation showed lower diversity.

Secondly, we picked nonsmoking, nondrinking dentate subjects from the original dataset to form a subset called the “unbiased data”. This subset consisted of 24 pre-treatment OSCC patients and 34 healthy controls who reported to not smoke tobacco or drink alcohol and who had at least one remaining tooth in their mouth. The microbial profiles of the two groups again differed statistically significantly (PERMANOVA, *F* = 2.4, *p* < 0.001), whereas there was no difference in the Shannon diversity index.

**Fig. 2:**
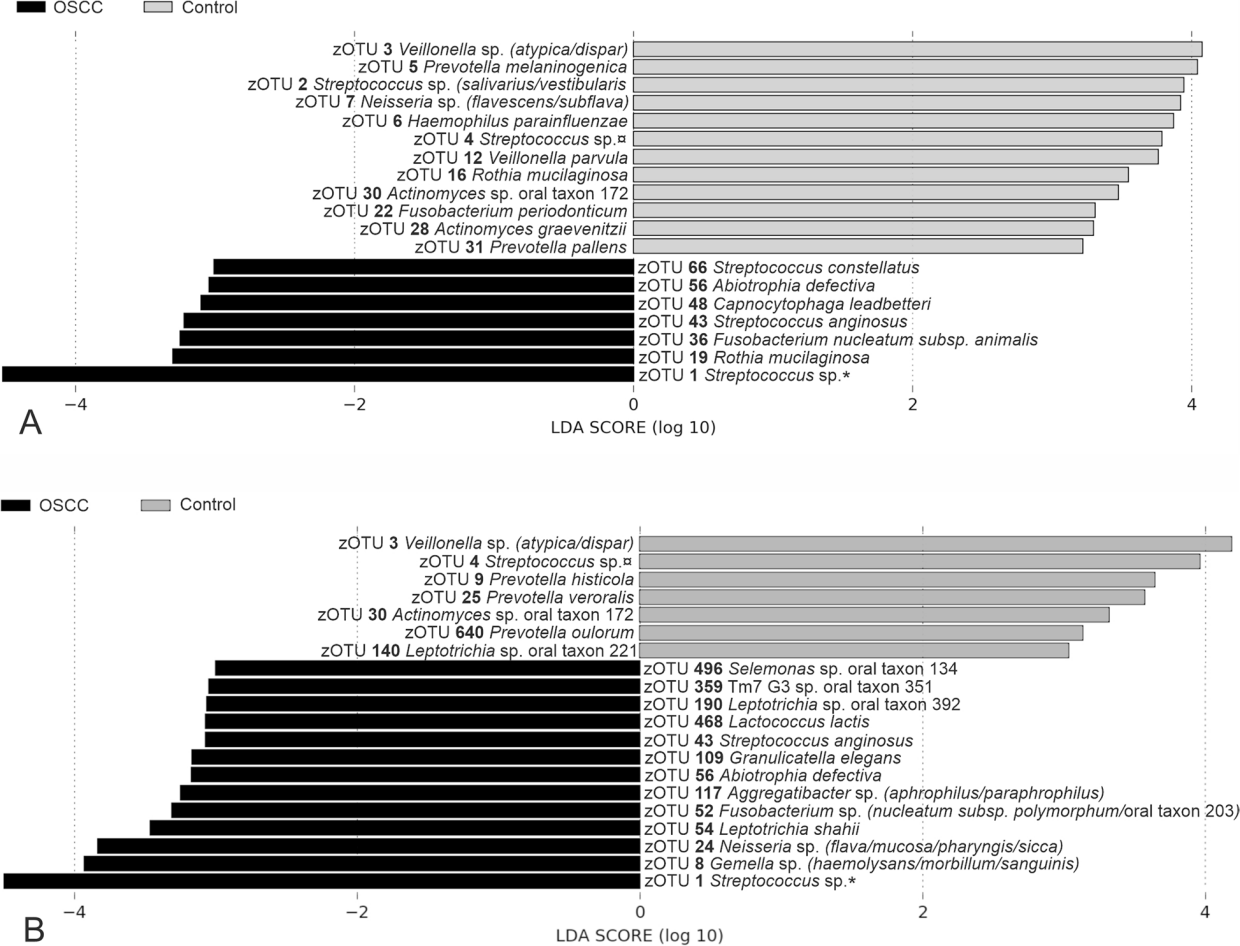
Discriminatory zOTUs between OSCC patients and healthy controls on biased **A** and unbiased **B** data. *zOTU1 *Streptococcusdentisani*/*infantis*/*mitis*/*oralis*/sp. oral taxon 058/sp. oral taxon 061/sp. oral taxon 064/sp. oral taxon 070/sp. oral taxon 423/sp. oral taxon 431/*tigurinus* (assigned taxonomy shortened in the figure). ^¤^zOTU4 *Streptococcusaustralis*/*parasanguinis* I/*parasanguinis* II/sp. oral taxon 057/sp. oral taxon 066 (assigned taxonomy shortened in the figure)

LEfSe-analysis was done for both the “biased” and the “unbiased” data (Fig. 2). In both datasets, significantly discriminatory zOTUs that were more abundant in the group OSCC included zOTU1 *Streptococcus dentisani/infantis/mitis/oralis/*oral_taxon_058*/*oral_taxon_061*/*oral_taxon_064*/*oral_taxon_070*/*oral_taxon_423* /*oral_taxon_431*/tigurinus*, zOTU43 *Streptococcus anginosus*, and zOTU56 *Abiotrophia defectiva*. The significant zOTUs more abundant in the control group included zOTU3 *Veillonella atypica/dispar*, zOTU4 *Streptococcus australis/parasanguinis_I/parasanguinis_II/*oral_taxon_057*/*oral_taxon_066, and zOTU30 *Actinomyces* oral_taxon_172.

In addition, the groups of zOTUs associated with either OSCC or the controls were disclosed by the Global Test (see Additional file 2). The Global Test produced 16 statistically significant groups of zOTUs—of which three consisted of a single zOTU—that were significantly distinguishing the patients from the controls (Additional file 3, sheet 1). Nine of the 13 groups of zOTUs were associated with OSCC (*p* = 0.012, *p* = 0.017, *p* = 0.025, *p* = 0.026, *p* = 0.047*, p* = 0.029*, p* = 0.033*, p* = 0.039, and *p* = 0.039) while three groups were associated with the controls (*p* = 0.025*, p* = 0.026, and *p* = 0.041). One group of zOTUs did not definitively associate with either the OSCC or the controls (*p* = 0.043). The most abundant genera within the OSCC-related groups of zOTUs were *Streptococcus*, *Fusobacterium*, *Capnocytophaga*, and *Lactobacillus*, and the most abundant genera within the control-related groups were *Prevotella*, *Actinomyces*, and *Megasphaera*. The three distinguishing zOTUs that were significant on their own were OSCC-related zOTU211 *Streptococcus anginosus* (*p* = 0.003), zOTU239 *Catonella morbi/*oral_taxon_164 (*p* = 0.007), and zOTU1 *Streptococcus dentisani/infantis/mitis/oralis/*oral_taxon_058*/*oral_taxon_061*/*oral_taxon_064*/*oral_taxon_070*/*oral_taxon_423* /*oral_taxon_431*/tigurinus* (*p* = 0.030).

To address the effect of smoking on the salivary microbiome, the OSCC and control groups were further divided according to the smoking status (Yes/No) and Global Test was used to disclose the groups of zOTUs associated with smoking and nonsmoking OSCC patients as well as smoking and nonsmoking controls. This time, the Global Test produced 19 significant groups of zOTUs that distinguished these four groups from one another (Additional file 3, sheet 2). Five of these were associated with the group OSCC smoker (all with *p* < 0.001) and four with OSCC nonsmoker (all with *p* < 0.001). Additionally, five zOTUs associated significantly with nonsmoker controls (all with *p* < 0.001) and only one group, consisting of a single zOTU, associated with smoker controls (*p* < 0.001). There were also four groups of zOTUs that were identified as significant but not associating to any of the specific groups. The most common genera among smoker OSCC patients were genus *Lactobacillus* and genus *Treponema*, whereas the only significant zOTU among smoker controls was zOTU 131 *Campylobacter rectus/showae*.

### Comparison of samples before and after cancer treatment

Samples from 28 OSCC patients were available after the cancer treatment at a mean follow-up period of 47.8 months (SD 12.1, min 35, max 78, Table 2). These 28 patients did not differ significantly at baseline from the other OSCC patients, except for having a higher prevalence of T1-tumors (Pearson chi-square, *p* = 0.035). At the follow-up, the SFR was significantly lower and the concentration of *Candida* in the saliva was higher than at baseline (paired samples *t* test *p* < 0.001 and related samples Wilcoxon signed-rank test *p* = 0.028, respectively).

**Table 2:** Basic characteristics of 28 OSCC patients analyzed before and after treatment

	Baseline	Follow-up	Difference between timepoints
Sex^a^, male (%)	15 (54%)		
Mean age, years (SD)	65.7 (10.3)	69.8 (10.3)	
Smokers (%)	11 (39%)		
Alcohol users (%)	20 (71%)		
Edentate (%)	1 (4%)	4 (14%)	NS
Mean SFR, ml/min (SD)	1.1 (0.6)	0.8 (0.6)	*p* = 0.0008
Dry mouth, SFR < 0.5 ml/min (%)	4 (14%)	10 (36%)	NS
Mean concertation of bacterial DNA, 16S qPCR, ng/μl (SD)	2.7 (2.3)	2.3 (2.7)	NS
Candidiasis^b^ (%)	11 (39%)	19 (68%)	*p* = 0.03
Mean *Candida* concentration, log10[CFU/ml] (SD)	2.3 (1.3)	2.9 (1.3)	NS
Mean concentration of yeasts, 28S qPCR, ng/μl (SD)	0.0049 (0.008)	0.0093 (0.02)	NS
Tumor class ^c^			
T1 (%)	19 (68%)		
T2–T4 (%)	9 (32%)		
Stage^c^			
I–II (%)	18 (64%)		
III–IV (%)	10 (36%)		
Treatment			
Excision with direct wound closure (%)	5 (28%)		
Reconstructive surgery (%)	11 (39%)		
Reconstructive surgery + radiation therapy (%)	11 (39%)		
Reconstructive surgery + chemoradiation therapy (%)	1 (4%)		

*SFR* salivary flow rate, *SD* standard deviation, *NS* non-significant

^a^Binary categorization determined using social security number

^b^*Candida* growth ≥ 400 colony-forming units (CFU) per milliliter of the saliva

^c^According to the 7th edition of the American Joint Committee on Cancer Staging Manual

**Fig. 3:**
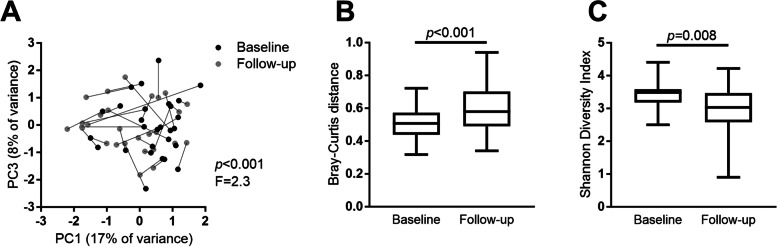
Comparison of the salivary microbiome from 28 OSCC patients before and after treatment. **A** Principal component analysis, *p* < 0.001, *F* = 2.3—restricted PERMANOVA. **B** Bray–Curtis distance, *p* < 0.001—Mann–Whitney test. **C** Shannon diversity index, *p* = 0.008—paired samples *t* test

The microbial profiles changed significantly from baseline to follow-up (restricted PERMANOVA, *F* = 2.3, *p* < 0.001, Fig. 3A), with the samples being less similar in composition (Bray–Curtis distance, Mann–Whitney *U* test, *p* < 0.001, Fig. 3B), and having lower alpha diversity (paired samples *t* test, *p* = 0.008, Fig. 3C) at the follow-up compared to the baseline. The microbial profiles of the OSCC patients at the follow-up were significantly different from those of the healthy controls (PERMANOVA, *F* = 6.43, *p* < 0.001), and the alpha diversity was significantly lower (Shannon diversity index, independent samples *t* test *p* = 0.003). The microbial profiles of the follow-up samples were significantly affected by hyposalivation (PERMANOVA, *F* = 1.711, *p* = 0.009), by having candidiasis (PERMANOVA, *F* = 1.983, *p* = 0.024), by having had loco-regionally advanced cancer (stage III or IV, PERMANOVA, *F* = 1.813, *p* = 0.036), and by having received adjuvant radiotherapy (PERMANOVA, *F* = 1.772, *p* = 0.041).

**Fig. 4:**
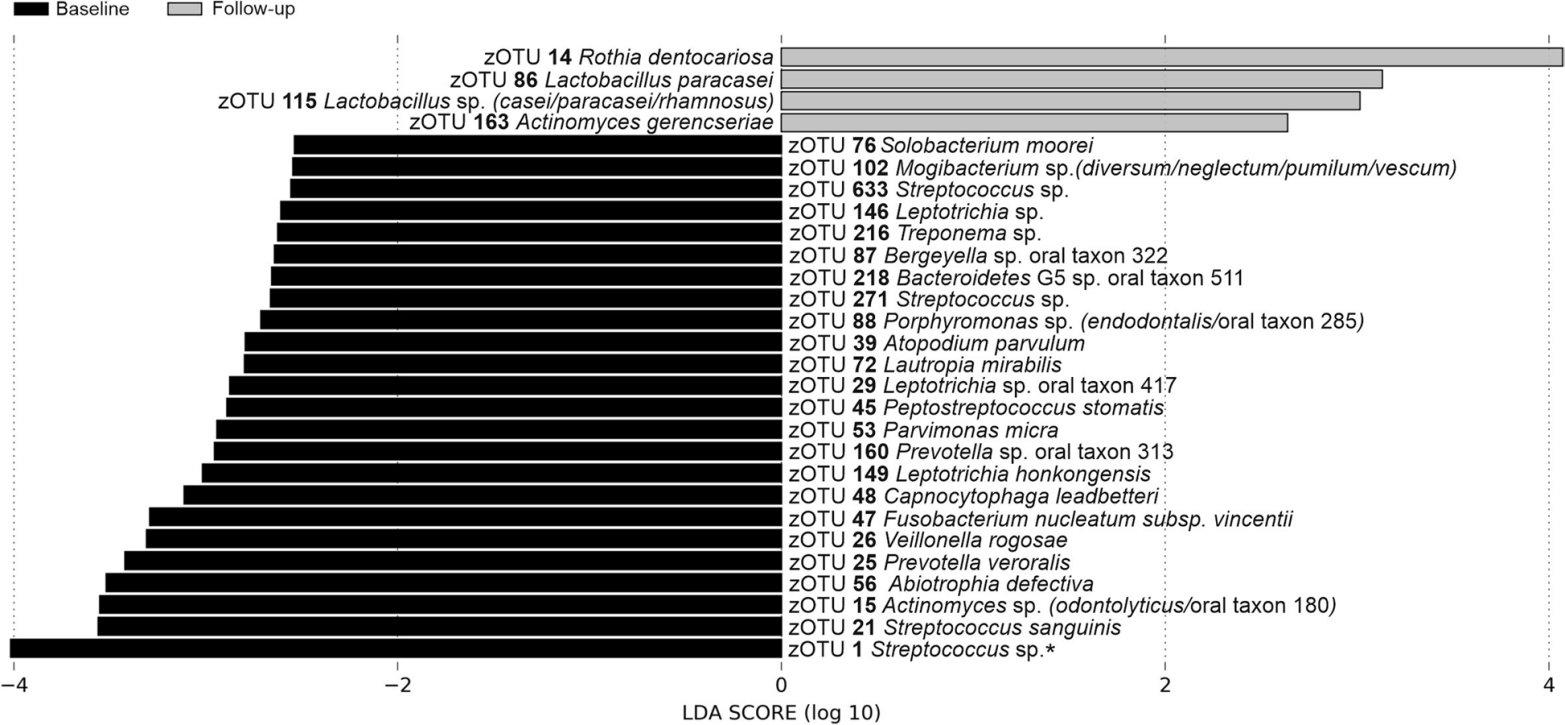
Discriminatory zOTUs between baseline and post-therapy samples in 28 OSCC patients (output of LEfSe analysis). *zOTU1 *Streptococcus dentisani*/*infantis*/*mitis*/*oralis*/sp. oral taxon 058/sp. oral taxon 061/sp. oral taxon 064/sp. oral taxon 070/sp. oral taxon 423/sp. oral taxon 431/*tigurinus* (assigned taxonomy shortened in the figure)

LEfSe-analysis identified 28 significantly discriminant zOTUs out of which 14 remained significant after FDR correction (Fig. 4). For the baseline samples, these were zOTU15 *Actinomyces odontolyticus/*oral_taxon_180, zOTU21 *Streptococcus sanguinis*, zOTU26 *Veillonella rogosae*, zOTU47 *Fusobacterium nucleatum vincentii*, zOTU48 *Capnocytophaga leadbetterii*, zOTU56 *Abiotrophia defectiva*, zOTU72 *Lautropia mirabilis*, zOTU76 *Solobacterium moorei*, zOTU88 *Porphyromonas endodontalis/*oral_taxon_285, zOTU102 *Mogibacterium diversum/neglectum/pumilum/vescum*, zOTU146 *Leptotrichia buccalis*, and zOTU633 *Streptococcus* sp. For the follow-up samples, the significantly associated zOTUs were zOTU115 *Lactobacillus casei/paracasei/rhamnosus* and zOTU163 *Actinomyces gerencseriae*.

## Discussion

The aim of this study was to investigate whether the salivary microbiome of OSCC patients is different from that of healthy individuals and how the microbiota changes from the pre-treatment to the post-treatment stage. We studied paraffin-wax-stimulated whole saliva samples of 99 OSCC patients prior to treatment and 28 post-treatment samples. These were compared with the results from 101 healthy controls. Based on these results, having OSCC seemed to have an effect on the salivary microbial profiles and the difference was evident even after removing the cofactors smoking, drinking, and being edentate. Cancer did not, however, have a significant effect on the microbial alpha diversity of the saliva samples.

The microbial profiles of the OSCC patients at baseline showed a higher relative abundance of bacteria previously linked to systemic infections, such as *S. anginosus*, *A. defectiva*, and *F. nucleatum*, whereas the microbial profiles of healthy controls had a higher abundance of bacteria previously associated with health, such as *Prevotella histicola*, *H. parainfluenzae*, and *F. periodonticum* [35–40]. *Streptococcus anginosus*, specifically, was shown to be significantly higher in abundance in the OSCC patients compared to the healthy controls by both LEfSe-analysis and the Global Test. In previous studies, *S. anginosus* has been reported to have caused bacteremia and related purulent infection foci in various organ systems [35]. Additionally, *A. defectiva*, which differentiated the OSCC patients at baseline from both their post-treatment samples and those of the healthy controls, has previously been linked to infective endocarditis and endophthalmitis [41, 42].

Species such as *F. nucleatum* and *C. leadbetteri*, which associate with the OSCC patients at baseline in our study, have also previously been linked to OSCC [13, 43]. *Porphyromonas gingivalis*, however, was not a discriminating factor between OSCC patients and healthy controls or the post-treatment samples in our data. This result was in contrast to earlier studies where a strong association was found between *P. gingivalis* and OSCC [12–15].

When the effect of smoking on the microbiome was tested using the Global Test, the most significant genera among smoker OSCC patients were genus *Lactobacillus* and genus *Treponema*. Specifically, the species related to smoker OSCC patients included several caries-related species such as *Lactobacillus gasseri* and *Lactobacillus fermentum* as well as *Treponema* species *T. socranskii* and *T. maltophilum*, which have been associated with severe periodontitis and periapical lesions [44–46]. This supports the link that has previously been shown between smoking, poor oral health, and OSCC [47].

After a relatively long follow-up time, the microbial profiles of the patients in the present series showed decreased similarity to each other and lower diversity within samples than at baseline. Furthermore, the microbial profiles were significantly different in the post-treatment samples when compared with those of the controls. The Shannon diversity index at the follow-up was significantly lower than that of the control samples. These results imply that the administered therapies indeed affect the salivary microbiome in the long term. This change may be unfavorable as the post-follow-up profile tended to lean towards an increase in aciduric taxa and a decrease in diversity, both of which have previously been linked to poor oral health [48, 49].

Eleven patients received adjuvant radiotherapy and one patient was treated with adjuvant chemoradiation therapy. At follow-up, there was a slight though significant difference in the microbial profiles between patients who had received radiotherapy and those who had not. Such difference was also evident when the follow-up samples were tested for the effect of having candidiasis, having hyposalivation and having had high stage cancer. Since patients with higher stage cancer tend to be treated with adjuvant (chemo)radiotherapy, which in turn is linked to both the development of hyposalivation and candidiasis, the difference found caused by the radiotherapy may actually be the result of one of the other factors found here to have a significant effect. In this study, the number of samples was too low to draw meaningful conclusions from this set of patients regarding the effect of radiotherapy. In previous studies, however, the effect of radiotherapy in head-and-neck cancer to the oral microbiome has been found to be profound and the increase in cariogenic taxa has been evident [50–52].

The postoperative oral health problems are among the principal complaints of OSCC patients [53]. Thus, the strive to establish a balanced oral microbiome post-OSCC-treatment should be seen as equally important as the efforts to re-establish function and appearance. As the microbial profiles of OSCC were significantly different from healthy controls both preoperatively and at follow-up, the oral health professionals should be encouraged to be more vocal about the need for balancing and stabilizing the oral microbiome. It should be noted, however, that the measures with which the positive modification of oral microbiome is done remain elusive and warrant for further research [54].

Finally, the weaknesses and strengths of the present study need to be discussed. The main weakness was the lack of full oral examination records, which, if completed with data on chewing capacity, saliva pH values and analysis on diet, would have been valuable for further conclusions. Especially the lack of full periodontal records from all participants causes a probable confounding factor that we could not correct for, as possible periodontal diseases are expected to affect the microbial profiles [55]. We were also unable to assess the effects of different treatment strategies on the results because of the small number of post-treatment follow-up samples. Furthermore, we were unable to match the controls by smoking, drinking, and edentulism. However, the percentages regarding smokers match the population statistics of the healthy population of Helsinki and Uusimaa district in Finland (8.1% of the population aged 65 and over in Helsinki and Uusimaa district in Finland, 2014 [56]).

The strength of this study is the homogeneity of the study group concerning age and Caucasian ethnicity in the Finnish population. The baseline study group of OSCC patients was also relatively large, which allowed us to study the microbial profiles both by excluding smokers, drinkers, and edentate subjects to form the unbiased group and by correcting for covariates with the Global Test. This made the statistical analyses more reliable, even though smoking could not be corrected for in the Global Test due to it leading to the loss of the effect of cancer in the analysis. A further strength was the relatively long follow-up time, which allowed us to investigate the long-term effects of OSCC therapy on the salivary microbiome.

In the future, we suggest additional studies involving the chemical composition of the saliva and its changes throughout the treatment of OSCC in order to account for any effects these changes may have on the salivary microbiome.

## Conclusions

Our study hypothesis was confirmed by showing that the microbial profiles in saliva indeed were different when comparing OSCC patients to healthy controls. Similarly, as we had expected, the post-treatment profiles differed significantly from those of the pre-treatment samples. The ecologically disadvantageous changes, seen as the increase in aciduric taxa and decrease of alpha diversity, as well as the higher relative abundance of potentially pathogenic taxa in the OSCC patients at baseline, are findings of clinical importance. Based on these results, we suggest that OSCC patients may benefit from preventive measures, such as recommendation of the use of pre- or probiotics, salivary substitutes, or dietary counseling. Furthermore, we recommend that the OSCC patients should have a longstanding relationship with the oral health professionals that extend beyond the 5-year follow-up with the surgical team in order to aid in the prevention of oral diseases after cancer treatment. All these could be expected to stabilize the oral microbiome, but intervention studies are needed for further conclusion in this area.

### Supplementary Information

**Additional file 1.** Alpha Diversity Indices - Detailed description and discussion of the alpha-diversity indices of the cross-sectional data examined during this study.

**Additional file 2.** Global Test Method - Detailed description of the statistical edgeR, residuals and the Global Test.

**Additional file 3.** Global Test Results (Excel spreadsheet, .xlsx) – Resulting groups of zOTUs from the Global Test.

## Data Availability

The datasets generated and/or analyzed during the current study are available in the NBCI BioProject repository, PRJNA997108 [https://www.ncbi.nlm.nih.gov/bioproject/997108].
