# Supplementary material for: Salivary microbiome profiles of oral cancer patients analyzed before and after treatment
Source: Microbiome. 2023 Aug 5;11:171. doi: 10.1186/s40168-023-01613-y (PMC10403937; doi:10.1186/s40168-023-01613-y)
Supplement: Supplementary file 2 — Additional file 1. Alpha Diversity Indices - Detailed description and discussion of the alpha-diversity indices of the cross-sectional data examined during this study. [file 40168_2023_1613_MOESM1_ESM.docx]

Additional File 1 of Mäkinen AI, Pappalardo VY, Buijs MJ, Brandt BW, Mäkitie AA, Meurman JH, Zaura E: Salivary microbiome profiles of oral cancer patients analysed before and after treatment.

**Alpha diversity indices**

Alpha diversity of the saliva samples was calculated in PAST software version 4.03 using untransformed data subsampled at 7900 reads/sample. The indices studied were the number of zOTUs (Taxa_S, Chao1, Shannon, and Simpson indices. The statistical significance of the differences of these values between OSCC patients and controls was tested using independent-samples t-test (Taxa_S, Chao1 and Shannon) or independent-samples Mann-Whitney U test (Simpson) on SPSS v.27 depending on normal distribution and equal variance of the data.

*Cross-sectional data*

Using the biased dataset (99 OSCC patients pre-treatment and 101 healthy controls), we found that the alpha diversity indices Taxa_S, Chao1 and Shannon did not statistically significantly differ between the OSCC patients and the healthy controls (Figure A1.1). The Simpson diversity index was, however, significantly different between the groups, with controls having higher values than the OSCC patients.


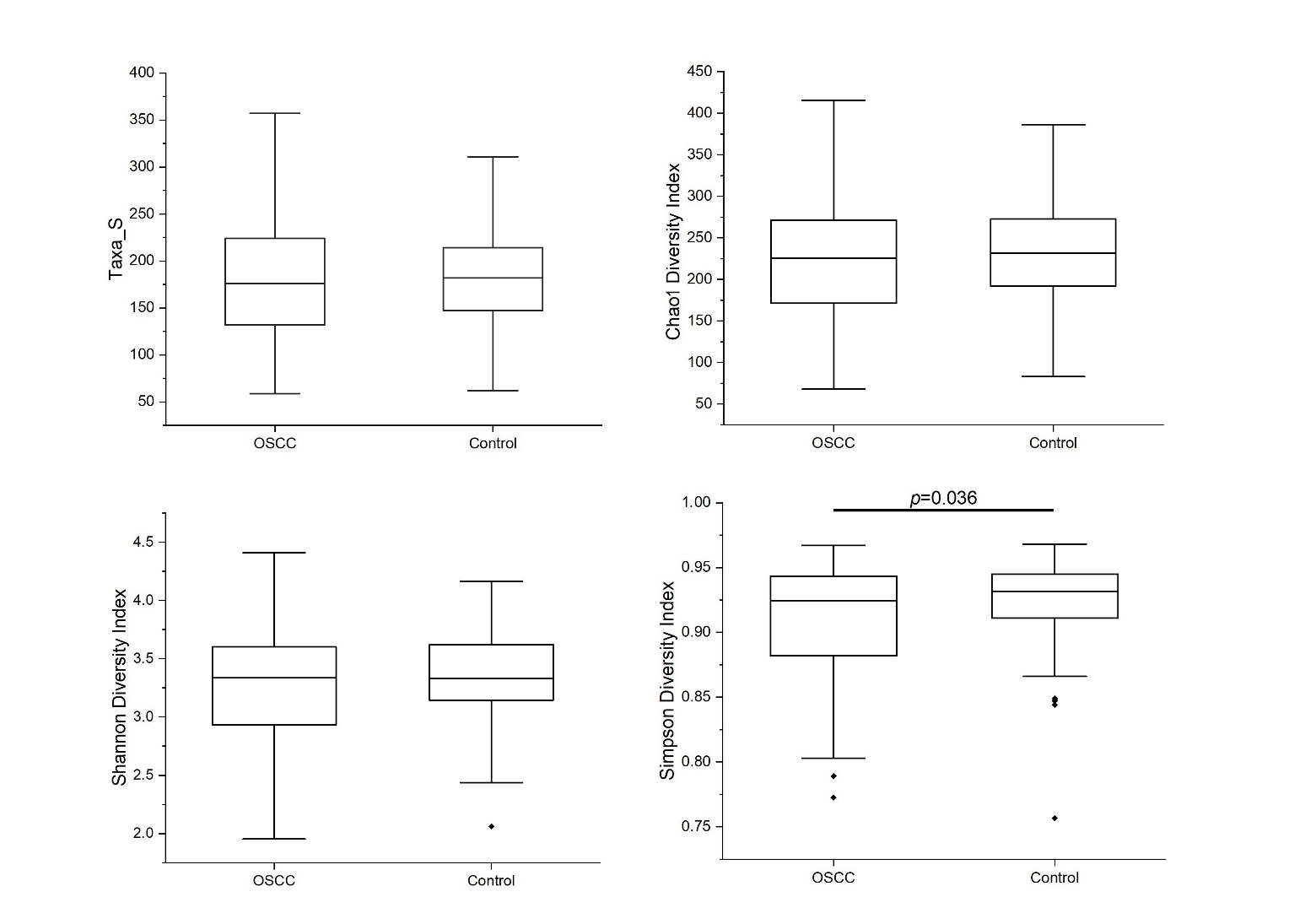


*Figure A1.1 Distribution of alpha diversity indices Taxa_S, Chao1, Shannon, and Simpson between OSCC patients and controls.*

After removing the smokers, drinkers and edentate subjects from the dataset (called the unbiased data), we found that none of the alpha diversity indices remained significant (*p*[Taxa]=0.214, *p*[Chao1]=0.210, *p*[Shannon]=0.940 Independent-samples t-test; *p*[Simpson]=0.641 Independent-samples Mann-Whitney U test).

*Discussion*

While the Simpson diversity index showed significant difference between the OSCC patients and the healthy controls in the biased data, we were unable to find significant differences in diversity using other indices or when using the unbiased data from which smokers, drinkers and edentate subjects were removed. The Simpson diversity index has been described to have a stronger sensitivity to species evenness, whereas the Shannon diversity is more sensitive to species richness [1]. The ability of this single index to pick-up significant difference where the other indices remain nonsignificant may, in this case, suggest that the difference in diversity is caused by the higher evenness of the zOTUs among the control patients. In the biased dataset, the distribution across all diversity indices was larger among OSCC patients than the healthy controls (Figure A1.1).

Based on these results, however, it would be difficult to conclude that the alpha diversity is significantly different between healthy controls and OSCC patients before treatment. In their commentary on microbiome diversity studies, Johnson and Burnet [1] call for caution when drawing conclusions from a single diversity index and argue that classic diversity indices may give an overly simplified view of the complicated microbiome health. Indeed, whereas a higher alpha diversity in the gut has been linked to higher stability and health, in the context of OSCC it has been associated with cancer lesions when compared to healthy tissue or saliva [2–4].
