# Supplementary material for: Salivary microbiome profiles of oral cancer patients analyzed before and after treatment
Source: Microbiome. 2023 Aug 5;11:171. doi: 10.1186/s40168-023-01613-y (PMC10403937; doi:10.1186/s40168-023-01613-y)
Supplement: Supplementary file 3 — Additional file 2. Global Test Method - Detailed description of the statistical edgeR, residuals and the Global Test. [file 40168_2023_1613_MOESM2_ESM.docx]

Additional file 2 of Mäkinen AI, Pappalardo VY, Buijs MJ, Brandt BW, Mäkitie AA, Meurman JH, Zaura E: Salivary microbiome profiles of oral cancer patients analysed before and after treatment.

**Detailed description of edgeR, residuals, and Global Test**

We aimed in this study to assess the relationship between cancer status and the salivary microbiome independently of clinical variables. To do so, the microbiome data was first corrected for the effect of clinical variables and subsequently tested for the association between the entire microbiome profile and cancer status.

First of all, the metadata file was cleaned by removing samples with uncertain responses or with missing values. This included all the samples from patients with values “Unknown” or “Casual” to the question about Smoking or “Unknown” to Alcohol habits or Edentate status. In total, 10 samples out of 200 were therefore excluded from the analysis. A total of eight covariates - remained for all 190 samples: Age, Sex, Smoking habit, Alcohol consumption, Edentate status, Saliva Flow Rate (SFR, ml/min), Candida concentration (log10, CFU/ml), and bacterial DNA concentration (16S rRNA gene, qPCR, ng/µl). The microbiome table was then filtered: zOTUs that appeared in less than 25% of the samples of one group (either Healthy or Disease) were removed from the analysis.

Subsequently, correction for the effect of clinical variables on microbiome by means of a generalized linear model was made. Specifically, a generalized linear model of the zOTU-table (response) was fitted on the clinical variables (explanatory variables). This is done using the package edgeR [1], which takes into account the nature of microbiome data, such as the zOTU counts being integers (reads per sample) and the data being over-dispersed, that is, the observed variance is larger than the expected one. The microbiome data after correction for the effect of the clinical variables corresponds to the standardized residuals of the fitted model.

Importantly, the effect of the variable ‘Smoking habit’ was not corrected for. Smoking was found to be the only variable significantly associated with the cancer status (Chi-square, Bonferroni-corrected *p*=1.25e-07). Therefore, the ‘Smoking habit’ could be a confounder of the cancer status, and for this reason, it was not corrected for.

To assess the relationship between the corrected microbiome and cancer status, the Global Test (globaltest package, version 5.44.0 [2,3]) with normal errors was used. For this reason, we first applied a hyperbolic arc sine transformation to the standardized residuals [4]. This transformation is equivalent to a logarithm for large values, and to a linear transformation for small values. Within the Global Test, correction for multiple testing is automatically performed using the inheritance method [5].

**Mathematical background**

One of the most common distributions to model the characteristics of microbiome data is the negative-binomial distribution. If *X_i,n_* ∈ **N**^+^ corresponds to the count of the *i*^th^ ∈ [1, *I*] OTU of the *n^th^* sample, we have:

$$X_{i,n}\mathcal{\sim NB}\left( \mu_{i},\phi_{i} \right)$$

where *µ_i_* ∈ **R**^+^ corresponds to the mean and *ϕ_i_* ∈ **R**^+^ to the dispersion parameters. With such a parametrization, we have:

$$f\left( x | \mu_{i},\phi_{i} \right)=\mathbf{P}\left( X=x \right)=\frac{\Gamma\left( x+\phi_{i}^{-1} \right)}{\Gamma\left( \phi_{i}^{-1} \right)\Gamma\left( x+1 \right)}\left( \frac{1}{1+\mu_{i}\phi_{i}} \right)^{\phi_{i}^{-1}}\left( \frac{\mu_{i}}{\mu_{i}+\phi_{i}^{-1}} \right)^{x},$$

$$\mathbf{E}\left( X_{i,n} \right)=\mu_{i},$$

$$\mathbf{V}\left( X_{i,n} \right)=\mu_{i}+\mu_{i}^{2}\phi_{i}.$$

In our case, we are interested in the impact of a set of covariates *Y_j,n_* on our microbiome *X_i,n_*. We, therefore, fitted a generalized linear model with *X_i,n_* modelled with a negative binomial distribution as a response, *Y_j,n_* as explanatory covariates, and *g : x → log(x)* as a link function. We then have ∀ *i*:

$$g\left( \mu_{i} \right)=Y_{j,n}\beta_{i,j}+\epsilon_{n}.$$

To fit this model, the glmFit function from the edgeR package (version 3.32.1) [1] was used. This function fits the model by computing the maximum likelihood estimator of the model. However, since the negative binomial distribution belongs to the exponential family only if the dispersion parameter is known, it first needs to be estimated. To do so, the estimateDisp function from the same package was used. This function uses an iterative method called Quantile Adjusted Conditional Maximum Likelihood to estimate *ϕ* and is explained in detail by Robinson et al [6].

To assess the relationship between cancer status and the salivary microbiome composition corrected for the effect of their covariates, we first computed the residuals *R_i,n_*:

$$R_{i,n}=X_{i,n}-Y_{j,n}\beta_{i,j.}$$

Then, we studied the standardized residuals after hyperbolic arc sine transformation:

$${SR}_{i,n}=\mathrm{arcsinh}\left( \frac{R_{i,n}}{\sqrt{Y_{j,n}\beta_{i,j}\left( 1+\phi_{i}Y_{j,n}\beta_{i,j} \right)}} \right).$$

We applied this transformation since it is the best one to bring the variance from being independent to the expectation which is a desirable property from the normal distribution [4].

Finally, those standardized residuals were used as explanatory variables in the Global Test. The Global Test is a statistical test able to assess the relationship between one variable of interest (here, the cancer status) and a group of variables (here, the residuals) even if the number of variables exceeds the number of samples. To achieve this, the Global Test first models the data with a classical generalized linear model. However, the effects of the covariates on our variables (represented by the regression coefficient *δ_i_*) is modelled as a random effect:

$$\delta_{i}\mathcal{\sim N}\left( 0,\sigma^{2} \right).$$

Test for the overall effect of our covariates correspond to:

$$H_{0}:\delta_{1}=\ldots=\delta_{I}=0,$$

which is equivalent to:

$$H_{0}^{'}:\sigma^{2}=0.$$

This hypothesis can be evaluated with the following test statistic:

$$Q=\frac{\left( Y-\mu\right)^{t}R\left( Y-\mu\right)}{\mu_{2}}\mathcal{\longrightarrow N}\left( 0, 1 \right),$$

with *R* = $\frac{1}{n}$*XX^t^*, *µ = g^−1^(β_0_), µ_2_* = **Ε**(*Y*^2^ | *H_0_*) and *Y* is our response.

**EdgeR and Global Test Results**

In this part of the study, we explored the relationship between OSCC cancer status, salivary microbiome, and covariates. The impact of the covariates on the salivary microbiome is visualized in Figure A2.1. The variable that has the largest impact is the edentate status. Being edentate indeed seemed to have an effect on several zOTUs. This is in line with studies that reported a decrease in diversity with the loss of teeth [7]. However, age, as well as bacterial DNA concentration, seemed to have little to no effect on microbiome composition.


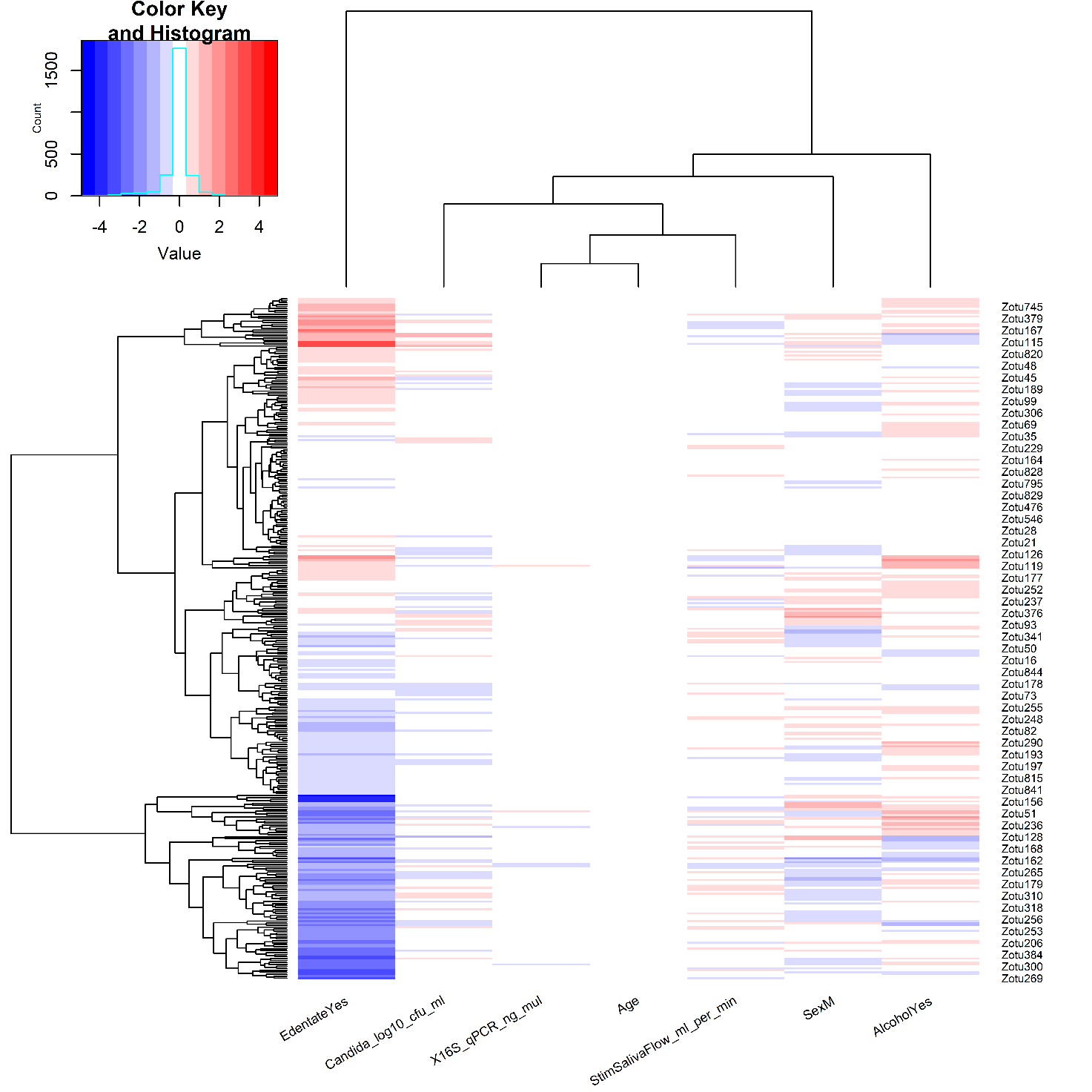


Figure A2.1: Heatmap of the regression coefficients for the covariates calculated by glmFit (edgeR). The x-axis shows the included covariates. The y-axis corresponds to the zOTUs. One coefficient is the impact of one specific covariate on a specific zOTUs. For example, being edentate decreased the abundance of zOTU269.

Figure A2.2 shows the zOTUs (or groups of zOTUs) that the global test found to be significantly associated with the cancer status. In total, 16 groups of zOTUs were significant (*p*=2.45e-09) and among them, three were composed of a single zOTU. While several genera – *Treponema*, *Streptococcus* or *Lactobacillus* – were found to associate with OSCC, it is more difficult to find taxa with a protective effect.


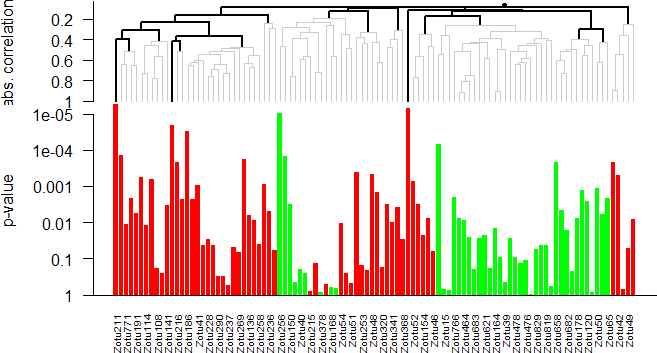


Figure A2.2: The dendrogram from the Global Test. The black lines in the dendrogram (top) indicate which groups of zOTUs are significantly associated with the cancer status. The higher a bar, the more significant the zOTU is associated to cancer status. Red corresponds to zOTUs associated with OSCC, while green corresponds to zOTUs associated with health.

After initially testing the associations with regard to groups OSCC and healthy controls, we divided the groups further according to smoking status: OSCC smokers (n=), OSCC non-smokers (n=), Healthy smokers (n=) and Healthy non-smokers (n=). This was done in order to address the profound effect smoking has on the oral microbiome . Figure A2.3 shows the groups of zOTUs that the global test found to be significantly associated with these four groups. Notably, there was only one group consisting of a single zOTU that was associated with the group Healthy smokers. This is most probably due to the small number of samples in this group.


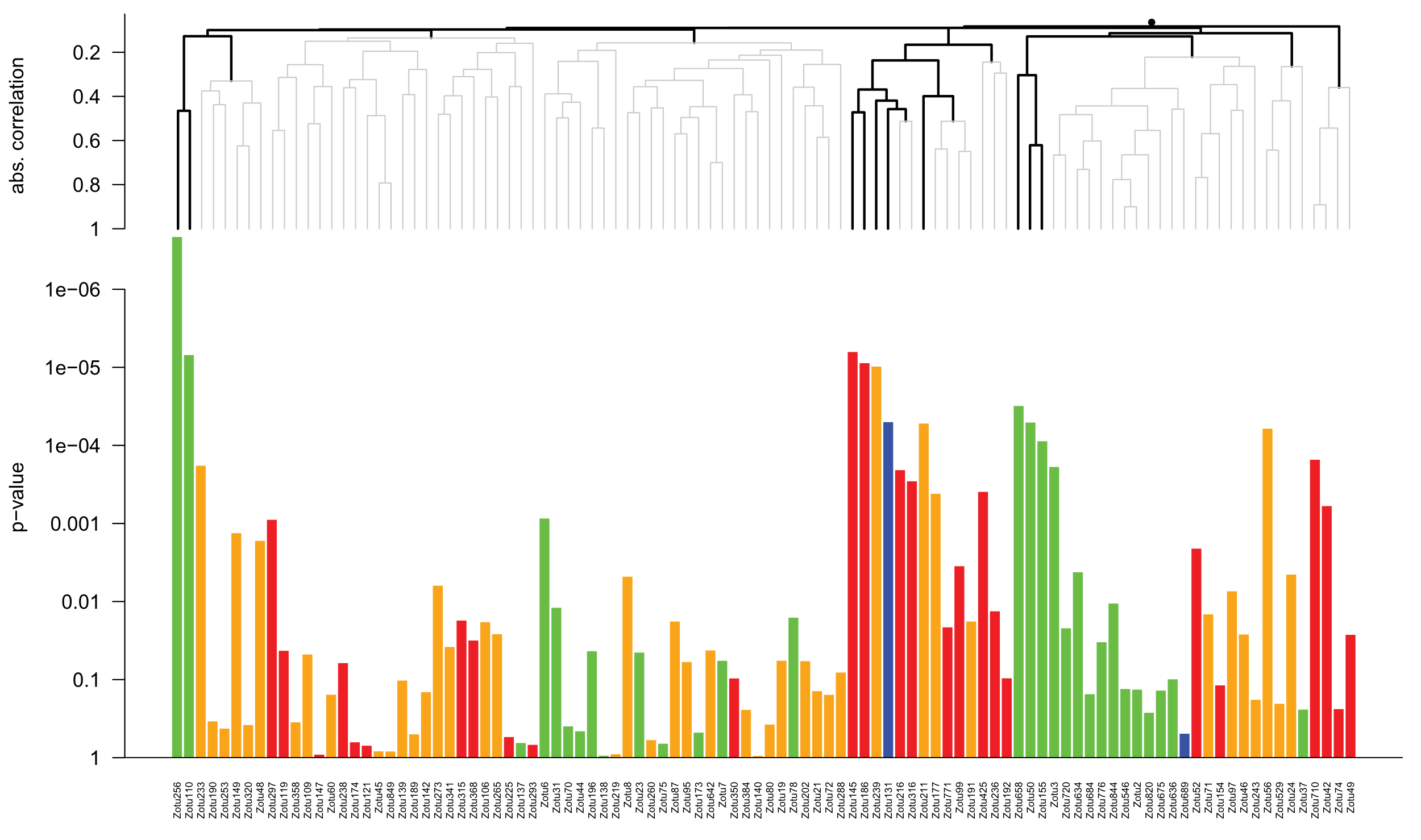


Figure A2.3: The dendrogram from the Global Test. The black lines in the dendrogram (top) indicate which groups of zOTUs are significantly associated with the cancer status. The higher a bar, the more significant the zOTU is associated to the group. Red corresponds to zOTUs associated with group OSCC smokers, orange corresponds to zOTUs associated with group OSCC non-smokers, blue corresponds to the zOTU associated with group Healthy smokers, and green corresponds to zOTUs associated with group Healthy non-smokers.

While the method we used here is a powerful and easy way to jointly analyse microbiome, covariates, and cancer status, it is important to notice that the negative-binomial distribution may not be the best to model microbiome data [8,9]. Indeed, the compositionality of our dataset is not taken into account by this model. Moreover, glmFit of edgeR tests each zOTU independently from other and thus the results lack a more ecological perspective. Another model which integrates both microbiome compositionality and zOTU correlation structure is still needed.

**References**

[1] Robinson MD, McCarthy DJ, Smyth GK. edgeR: a Bioconductor package for differential expression analysis of digital gene expression data. Bioinformatics 2010; doi:10.1093/bioinformatics/btp616.

[2] Goeman JJ, van de Geer SA, de Kort F, van Houwelingen HC. A global test for groups of genes: testing association with a clinical outcome. Bioinformatics 2004; doi:10.1093/bioinformatics/btg382.

[3] Goeman JJ, Oosting J. globaltest: Testing groups of covariates/features for association with a response variable, with applications to gene set testing. Bioconductor 2021; doi:/10.18129/B9.bioc.globaltest.

[4] Huber W, von Heydebreck A, Sültmann H, Poustka A, Vingron M. Variance stabilization applied to microarray data calibration and to the quantification of differential expression. Bioinformatics 2002; doi:10.1093/bioinformatics/18.suppl_1.S96.

[5] Goeman JJ, Finos L. The inheritance procedure: multiple testing of tree-structured hypotheses. Stat Appl Genet Mol Biol 2012; doi:10.1515/1544-6115.1554.

[6] Robinson MD, Smyth GK. Small-sample estimation of negative binomial dispersion, with applications to SAGE data. Biostatistics 2008; doi:10.1093/BIOSTATISTICS/KXM030.

[7] Gazdeck RK, Fruscione SR, Adami GR, Zhou Y, Cooper LF, Schwartz JL. Diversity of the oral microbiome between dentate and edentulous individuals. Oral Dis 2019; doi:10.1111/ODI.13039.

[8] Nearing JT, Douglas GM, Hayes MG, MacDonald J, Desai DK, Allward N, et al. Microbiome differential abundance methods produce different results across 38 datasets. Nat Commun 2022; doi:S41467-022-28034-Z.

[9] Weiss S, Xu ZZ, Peddada S, Amir A, Bittinger K, Gonzalez A, et al. Normalization and microbial differential abundance strategies depend upon data characteristics. Microbiome 2017; doi:10.1186/S40168-017-0237-Y.
